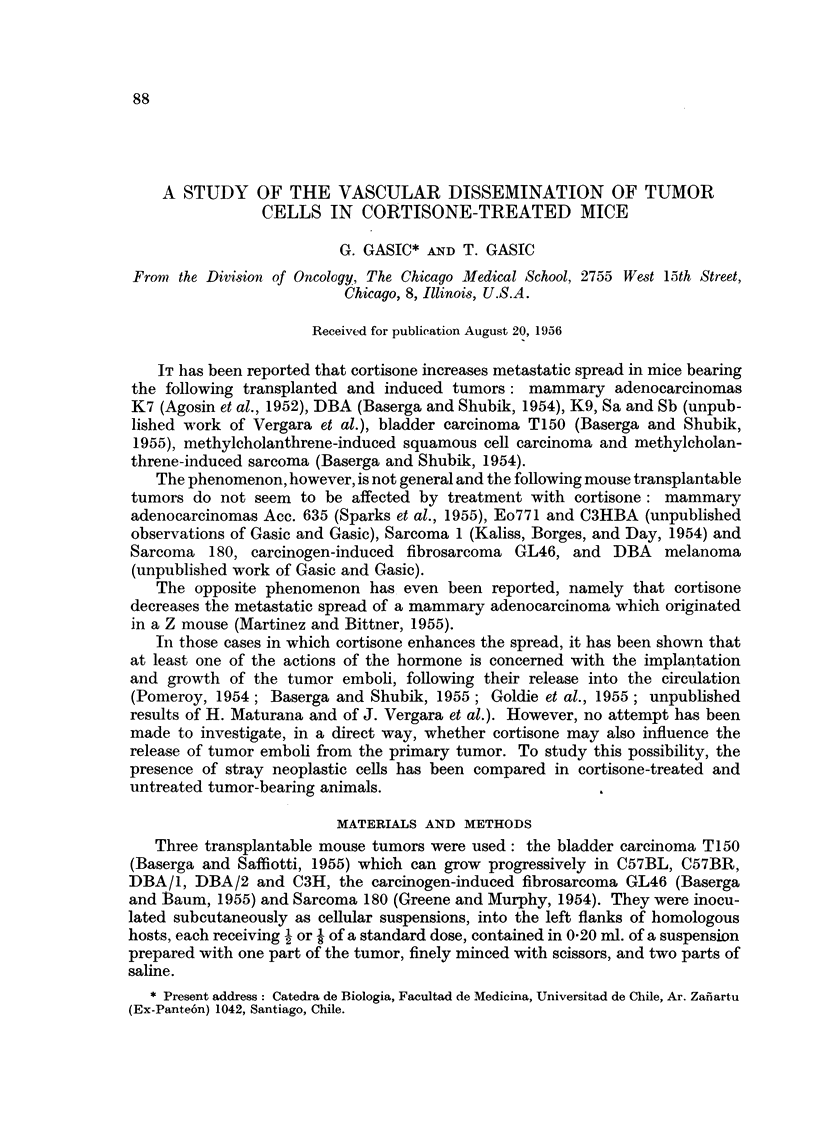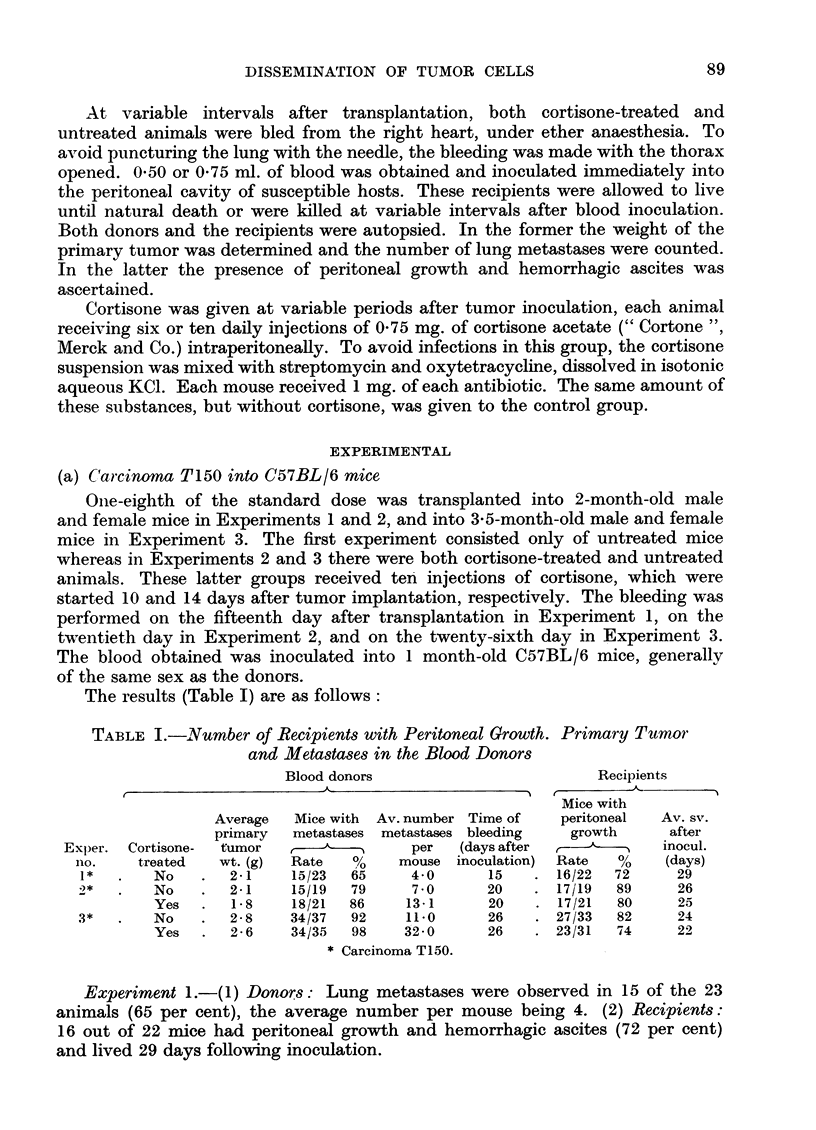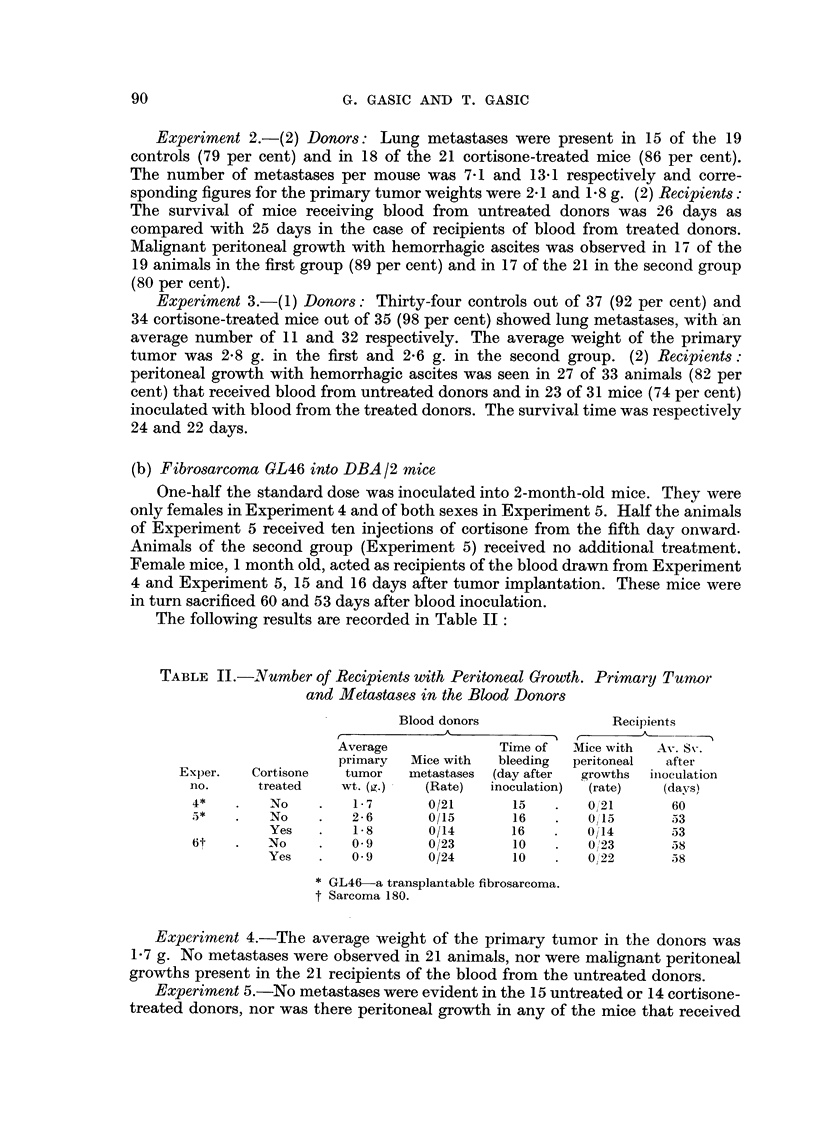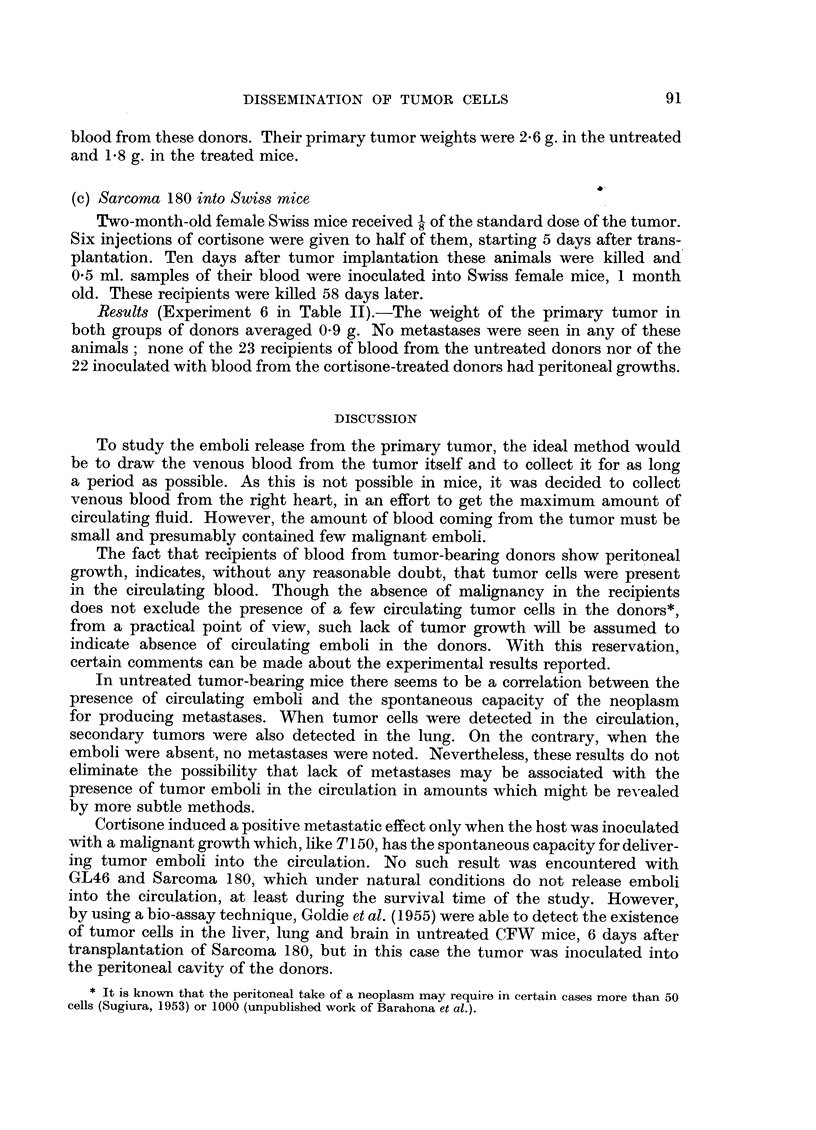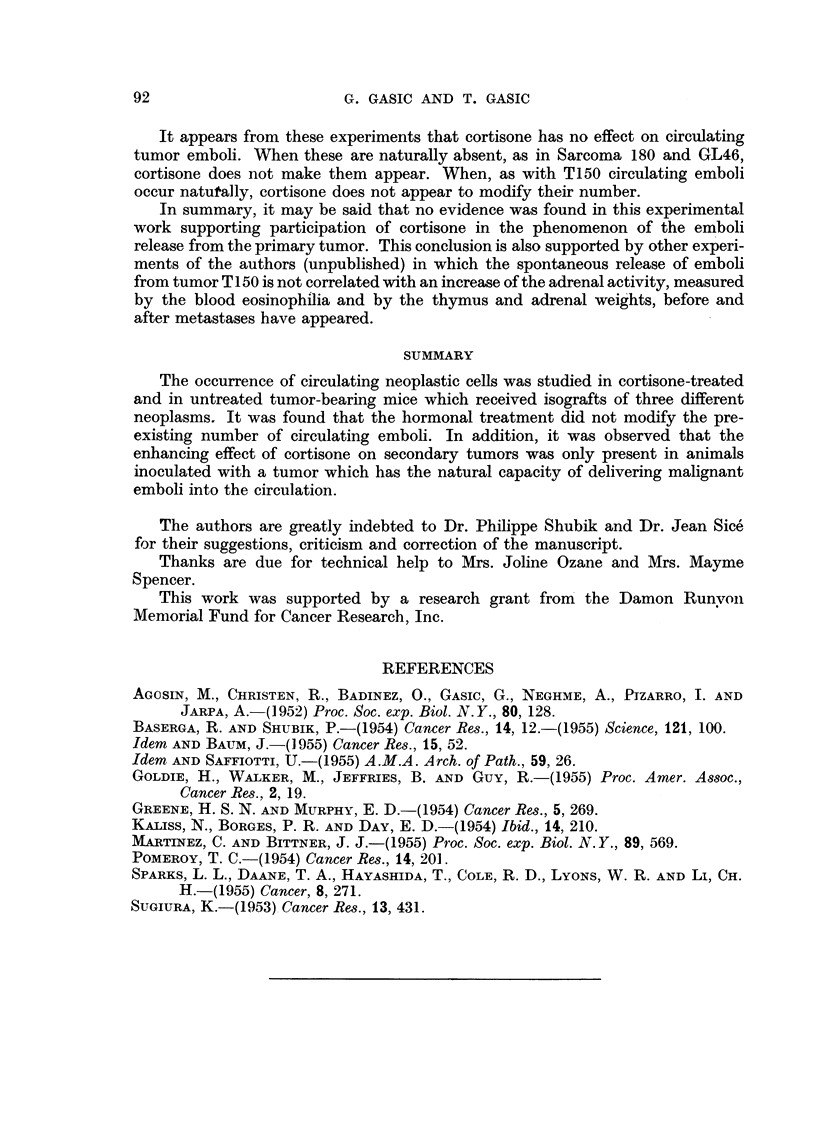# A Study of the Vascular Dissemination of Tumor Cells in Cortisone-Treated Mice

**DOI:** 10.1038/bjc.1957.13

**Published:** 1957-03

**Authors:** G. Gasic, T. Gasic


### A STUDY OF THE VASCULAR DISSEMINATION OF TUMOR CELLS IN CORTISONE-TREATED MICE

G. GASIC* AND T. GASIC

*From the Division of Oncology, The Chicago Medical School,* 2755 *West* 15*th Street, Chicago,* 8, *Illinois, U.S.A.*

Received for publication August 20, 1956

It has been reported that cortisone increases metastatic spread in mice bearing the following transplanted and induced tumors : mammary adenocarcinomas K7 (Agosin *et al.*, 1952), DBA (Baserga and Shubik, 1954), K9, Sa and Sb (unpublished work of Vergara *et al.*), bladder carcinoma T150 (Baserga and Shubik, 1955), methylcholanthrene-induced squamous cell carcinoma and methylcholanthrene-induced sarcoma (Baserga and Shubik, 1954).

The phenomenon, however, is not general and the following mouse transplantable tumors do not seem to be affected by treatment with cortisone : mammary adenocarcinomas Acc. 635 (Sparks *et al.*, 1955), Eo771 and C3HBA (unpublished observations of Gasic and Gasic), Sarcoma 1 (Kaliss, Borges, and Day, 1954) and Sarcoma 180, carcinogen-induced fibrosarcoma GL46, and DBA melanoma (unpublished work of Gasic and Gasic).

The opposite phenomenon has even been reported, namely that cortisone decreases the metastatic spread of a mammary adenocarcinoma which originated in a Z mouse (Martinez and Bittner, 1955).

In those cases in which cortisone enhances the spread, it has been shown that at least one of the actions of the hormone is concerned with the implantation and growth of the tumor emboli, following their release into the circulation (Pomeroy, 1954 ; Baserga and Shubik, 1955 ; Goldie *et al.*, 1955 ; unpublished results of H. Maturana and of J. Vergara *et al.*). However, no attempt has been made to investigate, in a direct way, whether cortisone may also influence the release of tumor emboli from the primary tumor. To study this possibility, the presence of stray neoplastic cells has been compared in cortisone-treated and untreated tumor-bearing animals.

### MATERIALS AND METHODS

Three transplantable mouse tumors were used : the bladder carcinoma T150 (Baserga and Saffiotti, 1955) which can grow progressively in C57BL, C57BR, DBA/1, DBA/2 and C3H, the carcinogen-induced fibrosarcoma GL46 (Baserga and Baum, 1955) and Sarcoma 180 (Greene and Murphy, 1954). They were inoculated subcutaneously as cellular suspensions, into the left flanks of homologous hosts, each receiving $\frac{1}{2}$ or $\frac{1}{8}$ of a standard dose, contained in 0·20 ml. of a suspension prepared with one part of the tumor, finely minced with scissors, and two parts of saline.

* Present address : Catedra de Biologia, Facultad de Medicina, Universitad de Chile, Ar. Zañartu (Ex-Panteón) 1042, Santiago, Chile.

At variable intervals after transplantation, both cortisone-treated and untreated animals were bled from the right heart, under ether anaesthesia. To avoid puncturing the lung with the needle, the bleeding was made with the thorax opened. 0·50 or 0·75 ml. of blood was obtained and inoculated immediately into the peritoneal cavity of susceptible hosts. These recipients were allowed to live until natural death or were killed at variable intervals after blood inoculation. Both donors and the recipients were autopsied. In the former the weight of the primary tumor was determined and the number of lung metastases were counted. In the latter the presence of peritoneal growth and hemorrhagic ascites was ascertained.

Cortisone was given at variable periods after tumor inoculation, each animal receiving six or ten daily injections of 0·75 mg. of cortisone acetate (" Cortone ", Merck and Co.) intraperitoneally. To avoid infections in this group, the cortisone suspension was mixed with streptomycin and oxytetracycline, dissolved in isotonic aqueous KCl. Each mouse received 1 mg. of each antibiotic. The same amount of these substances, but without cortisone, was given to the control group.

### EXPERIMENTAL

### (a) *Carcinoma T150 into C57BL/6 mice*

One-eighth of the standard dose was transplanted into 2-month-old male and female mice in Experiments 1 and 2, and into 3·5-month-old male and female mice in Experiment 3. The first experiment consisted only of untreated mice whereas in Experiments 2 and 3 there were both cortisone-treated and untreated animals. These latter groups received ten injections of cortisone, which were started 10 and 14 days after tumor implantation, respectively. The bleeding was performed on the fifteenth day after transplantation in Experiment 1, on the twentieth day in Experiment 2, and on the twenty-sixth day in Experiment 3. The blood obtained was inoculated into 1 month-old C57BL/6 mice, generally of the same sex as the donors.

The results (Table I) are as follows :

TABLE I.—*Number of Recipients with Peritoneal Growth. Primary Tumor and Metastases in the Blood Donors*

| | Blood donors | | | | | | Recipients | | |
|---|---|---|---|---|---|---|---|---|---|
| | | | Mice with metastases | | | | Mice with peritoneal growth | | |
| Exper. no. | Cortisone-treated | Average primary tumor wt. (g) | Rate | % | Av. number metastases per mouse | Time of bleeding (days after inoculation) | Rate | % | Av. sv. after inocul. (days) |
| 1* | No | 2·1 | 15/23 | 65 | 4·0 | 15 | 16/22 | 72 | 29 |
| 2* | No | 2·1 | 15/19 | 79 | 7·0 | 20 | 17/19 | 89 | 26 |
| | Yes | 1·8 | 18/21 | 86 | 13·1 | 20 | 17/21 | 80 | 25 |
| 3* | No | 2·8 | 34/37 | 92 | 11·0 | 26 | 27/33 | 82 | 24 |
| | Yes | 2·6 | 34/35 | 98 | 32·0 | 26 | 23/31 | 74 | 22 |

* Carcinoma T150.

*Experiment* 1.—(1) *Donors:* Lung metastases were observed in 15 of the 23 animals (65 per cent), the average number per mouse being 4. (2) *Recipients:* 16 out of 22 mice had peritoneal growth and hemorrhagic ascites (72 per cent) and lived 29 days following inoculation.

*Experiment* 2.—(2) *Donors:* Lung metastases were present in 15 of the 19 controls (79 per cent) and in 18 of the 21 cortisone-treated mice (86 per cent). The number of metastases per mouse was 7·1 and 13·1 respectively and corresponding figures for the primary tumor weights were 2·1 and 1·8 g. (2) *Recipients:* The survival of mice receiving blood from untreated donors was 26 days as compared with 25 days in the case of recipients of blood from treated donors. Malignant peritoneal growth with hemorrhagic ascites was observed in 17 of the 19 animals in the first group (89 per cent) and in 17 of the 21 in the second group (80 per cent).

*Experiment* 3.—(1) *Donors:* Thirty-four controls out of 37 (92 per cent) and 34 cortisone-treated mice out of 35 (98 per cent) showed lung metastases, with an average number of 11 and 32 respectively. The average weight of the primary tumor was 2·8 g. in the first and 2·6 g. in the second group. (2) *Recipients:* peritoneal growth with hemorrhagic ascites was seen in 27 of 33 animals (82 per cent) that received blood from untreated donors and in 23 of 31 mice (74 per cent) inoculated with blood from the treated donors. The survival time was respectively 24 and 22 days.

(b) *Fibrosarcoma GL46 into DBA/2 mice*

One-half the standard dose was inoculated into 2-month-old mice. They were only females in Experiment 4 and of both sexes in Experiment 5. Half the animals of Experiment 5 received ten injections of cortisone from the fifth day onward. Animals of the second group (Experiment 5) received no additional treatment. Female mice, 1 month old, acted as recipients of the blood drawn from Experiment 4 and Experiment 5, 15 and 16 days after tumor implantation. These mice were in turn sacrificed 60 and 53 days after blood inoculation.

The following results are recorded in Table II :

TABLE II.—*Number of Recipients with Peritoneal Growth. Primary Tumor and Metastases in the Blood Donors*

| | | Blood donors | | | Recipients | |
|---|---|---|---|---|---|---|
| Exper. no. | Cortisone treated | Average primary tumor wt. (g.) | Mice with metastases (Rate) | Time of bleeding (day after inoculation) | Mice with peritoneal growths (rate) | Av. Sv. after inoculation (days) |
| 4* | No | 1·7 | 0/21 | 15 | 0/21 | 60 |
| 5* | No | 2·6 | 0/15 | 16 | 0/15 | 53 |
| | Yes | 1·8 | 0/14 | 16 | 0/14 | 53 |
| 6† | No | 0·9 | 0/23 | 10 | 0/23 | 58 |
| | Yes | 0·9 | 0/24 | 10 | 0/22 | 58 |

* GL46—a transplantable fibrosarcoma.

† Sarcoma 180.

*Experiment* 4.—The average weight of the primary tumor in the donors was 1·7 g. No metastases were observed in 21 animals, nor were malignant peritoneal growths present in the 21 recipients of the blood from the untreated donors.

*Experiment* 5.—No metastases were evident in the 15 untreated or 14 cortisone-treated donors, nor was there peritoneal growth in any of the mice that received blood from these donors. Their primary tumor weights were 2·6 g. in the untreated and 1·8 g. in the treated mice.

(c) *Sarcoma* 180 *into Swiss mice*

Two-month-old female Swiss mice received $\frac{1}{8}$ of the standard dose of the tumor. Six injections of cortisone were given to half of them, starting 5 days after transplantation. Ten days after tumor implantation these animals were killed and 0·5 ml. samples of their blood were inoculated into Swiss female mice, 1 month old. These recipients were killed 58 days later.

*Results* (Experiment 6 in Table II).—The weight of the primary tumor in both groups of donors averaged 0·9 g. No metastases were seen in any of these animals ; none of the 23 recipients of blood from the untreated donors nor of the 22 inoculated with blood from the cortisone-treated donors had peritoneal growths.

### DISCUSSION

To study the emboli release from the primary tumor, the ideal method would be to draw the venous blood from the tumor itself and to collect it for as long a period as possible. As this is not possible in mice, it was decided to collect venous blood from the right heart, in an effort to get the maximum amount of circulating fluid. However, the amount of blood coming from the tumor must be small and presumably contained few malignant emboli.

The fact that recipients of blood from tumor-bearing donors show peritoneal growth, indicates, without any reasonable doubt, that tumor cells were present in the circulating blood. Though the absence of malignancy in the recipients does not exclude the presence of a few circulating tumor cells in the donors*, from a practical point of view, such lack of tumor growth will be assumed to indicate absence of circulating emboli in the donors. With this reservation, certain comments can be made about the experimental results reported.

In untreated tumor-bearing mice there seems to be a correlation between the presence of circulating emboli and the spontaneous capacity of the neoplasm for producing metastases. When tumor cells were detected in the circulation, secondary tumors were also detected in the lung. On the contrary, when the emboli were absent, no metastases were noted. Nevertheless, these results do not eliminate the possibility that lack of metastases may be associated with the presence of tumor emboli in the circulation in amounts which might be revealed by more subtle methods.

Cortisone induced a positive metastatic effect only when the host was inoculated with a malignant growth which, like *T*150, has the spontaneous capacity for delivering tumor emboli into the circulation. No such result was encountered with GL46 and Sarcoma 180, which under natural conditions do not release emboli into the circulation, at least during the survival time of the study. However, by using a bio-assay technique, Goldie *et al.* (1955) were able to detect the existence of tumor cells in the liver, lung and brain in untreated CFW mice, 6 days after transplantation of Sarcoma 180, but in this case the tumor was inoculated into the peritoneal cavity of the donors.

* It is known that the peritoneal take of a neoplasm may require in certain cases more than 50 cells (Sugiura, 1953) or 1000 (unpublished work of Barahona *et al.*).

It appears from these experiments that cortisone has no effect on circulating tumor emboli. When these are naturally absent, as in Sarcoma 180 and GL46, cortisone does not make them appear. When, as with T150 circulating emboli occur naturally, cortisone does not appear to modify their number.

In summary, it may be said that no evidence was found in this experimental work supporting participation of cortisone in the phenomenon of the emboli release from the primary tumor. This conclusion is also supported by other experiments of the authors (unpublished) in which the spontaneous release of emboli from tumor T150 is not correlated with an increase of the adrenal activity, measured by the blood eosinophilia and by the thymus and adrenal weights, before and after metastases have appeared.

### SUMMARY

The occurrence of circulating neoplastic cells was studied in cortisone-treated and in untreated tumor-bearing mice which received isografts of three different neoplasms. It was found that the hormonal treatment did not modify the preexisting number of circulating emboli. In addition, it was observed that the enhancing effect of cortisone on secondary tumors was only present in animals inoculated with a tumor which has the natural capacity of delivering malignant emboli into the circulation.

The authors are greatly indebted to Dr. Philippe Shubik and Dr. Jean Sicé for their suggestions, criticism and correction of the manuscript.

Thanks are due for technical help to Mrs. Joline Ozane and Mrs. Mayme Spencer.

This work was supported by a research grant from the Damon Runyon Memorial Fund for Cancer Research, Inc.

### REFERENCES

AGOSIN, M., CHRISTEN, R., BADINEZ, O., GASIC, G., NEGHME, A., PIZARRO, I. AND JARPA, A.—(1952) *Proc. Soc. exp. Biol. N.Y.*, **80**, 128.

BASERGA, R. AND SHUBIK, P.—(1954) *Cancer Res.*, **14**, 12.—(1955) *Science*, **121**, 100.

*Idem* AND BAUM, J.—(1955) *Cancer Res.*, **15**, 52.

*Idem* AND SAFFIOTTI, U.—(1955) *A.M.A. Arch. of Path.*, **59**, 26.

GOLDIE, H., WALKER, M., JEFFRIES, B. AND GUY, R.—(1955) *Proc. Amer. Assoc., Cancer Res.*, **2**, 19.

GREENE, H. S. N. AND MURPHY, E. D.—(1954) *Cancer Res.*, **5**, 269.

KALISS, N., BORGES, P. R. AND DAY, E. D.—(1954) *Ibid.*, **14**, 210.

MARTINEZ, C. AND BITTNER, J. J.—(1955) *Proc. Soc. exp. Biol. N.Y.*, **89**, 569.

POMEROY, T. C.—(1954) *Cancer Res.*, **14**, 201.

SPARKS, L. L., DAANE, T. A., HAYASHIDA, T., COLE, R. D., LYONS, W. R. AND LI, CH. H.—(1955) *Cancer*, **8**, 271.

SUGIURA, K.—(1953) *Cancer Res.*, **13**, 431.